# Intra- and inter-radiation therapist reproducibility of daily isocenter verification using prostatic fiducial markers

**DOI:** 10.1186/1748-717X-1-2

**Published:** 2006-02-28

**Authors:** Karen L Ullman, Holly Ning, Robert C Susil, Asna Ayele, Lucresse Jocelyn, Jan Havelos, Peter Guion, Huchen Xie, Guang Li, Barbara C Arora, Angela Cannon, Robert W Miller, C Norman Coleman, Kevin Camphausen, Cynthia Ménard

**Affiliations:** 1 Radiation Oncology Branch, Center for Cancer Research, National Cancer Institute, NIH, DHHS, Bldg 10, CRC Rm B2(SW) 3500, 9000 Rockville Pike, Bethesda, MD, 20892, USA; 2 Department of Biomedical Engineering, Johns Hopkins University School of Medicine, 720 Rutland Ave, Baltimore, Maryland, 21205, USA; 3 Radiation Medicine Program, University of Toronto, Princess Margaret Hospital, 610 University Avenue, Toronto, Ontario, M4X 1C3, Canada

## Abstract

**Background:**

We sought to determine the intra- and inter-radiation therapist reproducibility of a previously established matching technique for daily verification and correction of isocenter position relative to intraprostatic fiducial markers (FM).

**Materials and methods:**

With the patient in the treatment position, anterior-posterior and left lateral electronic images are acquired on an amorphous silicon flat panel electronic portal imaging device. After each portal image is acquired, the therapist manually translates and aligns the fiducial markers in the image to the marker contours on the digitally reconstructed radiograph. The distances between the planned and actual isocenter location is displayed. In order to determine the reproducibility of this technique, four therapists repeated and recorded this operation two separate times on 20 previously acquired portal image datasets from two patients. The data were analyzed to obtain the mean variability in the distances measured between and within observers.

**Results:**

The mean and median intra-observer variability ranged from 0.4 to 0.7 mm and 0.3 to 0.6 mm respectively with a standard deviation of 0.4 to 1.0 mm. Inter-observer results were similar with a mean variability of 0.9 mm, a median of 0.6 mm, and a standard deviation of 0.7 mm. When using a 5 mm threshold, only 0.5% of treatments will undergo a table shift due to intra or inter-observer error, increasing to an error rate of 2.4% if this threshold were reduced to 3 mm.

**Conclusion:**

We have found high reproducibility with a previously established method for daily verification and correction of isocenter position relative to prostatic fiducial markers using electronic portal imaging.

## Introduction

Carcinoma of the prostate is the most common form of cancer in men [1]. In 2005, 232,090 new cases of prostate cancer and 30,350 prostate cancer deaths were projected in the United States [1]. External beam radiotherapy constitutes one of the mainstays of therapy for patients with localized disease. Given the relatively small treatment fields used with conformal and intensity-modulated radiotherapy, there is a greater need for accurate targeting and daily localization of the prostate gland, a task is being performed by radiation therapists/technologists.

The prostate is located between the bladder and the rectum, and its position within the pelvis can change according to the degree of fullness of the bladder and rectum. Variations in bladder and rectal filling have been shown to affect prostate position within the pelvis, to an extent which may require field adjustments during the course of radiotherapy [2]. Since the rectum tends to become progressively less distended during a course of pelvic radiotherapy (mean decrease in diameter 1.5 cm), the predominant prostate motion is in the posterior and inferior direction. In one study, 11% of patients showed an inferior shift of the prostate of more than 1 cm and 30% showed a posterior shift of more than 1 cm [3]. Indeed, this variation in position cannot be assessed with external skin marks or bony landmarks, the standard approach used by radiation therapists on a daily basis. Any motion of the target relative to these landmarks limits the accuracy of radiotherapy. A safety margin is therefore applied during treatment planning to ensure correct irradiation of the target in spite of this movement.

Portal x-ray imaging is a technique used to monitor the accuracy of beam isocenter positioning relative to bony landmarks or fiducial markers during radiotherapy. Since the prostate is not visible on portal imaging, radiopaque fiducial markers are surrogates for organ localization in portal images [4]. As prostate motion is the major source of error in radiation treatment delivery [5], some investigators have recommended that radio-opaque markers be placed in the prostate prior to the start of radiotherapy. In our clinic, a previously described technique for daily electronic portal imaging device (EPID) visualization and alignment to prostate fiducial markers has been implemented to reduce inter-fractional set-up uncertainty, with the eventual goal of safely reducing PTV margins and normal tissue dose [6]. In order to determine, in part, the targeting error associated with this technique, we sought to measure intra and inter-radiation therapist variability using fiducial markers for daily set-up assessment and adjustment of external radiation beam targeting.

## Methods and materials

### Fiducial marker placement under MRI-guidance

The patient subjects of this study were enrolled on an IRB approved protocol after providing informed consent. The primary objective of this protocol was to validate the accuracy and tolerability of a new device that allows for the placement of needles and fiducial markers within the prostate gland based upon MR images instead of standard ultrasound images. The secondary objective was to gain experience using fiducial markers for daily assessment and adjustment of external radiation beam targeting as performed by radiation therapists.

Four sterile gold fiducial markers (1.2 × 3 mm, Med Tec^® ^– NWMP, Iowa) are placed within the prostate under MRI guidance one week before external beam radiotherapy in patients with localized prostate cancer [7]. Markers are placed at the prostate base, apex, and right and left margins at the level of the mid-gland. The patient returns four to five days later for a treatment planning MRI and a treatment planning non-contrast CT.

#### Treatment planning

Treatment planning MRI consists of a T2-weighted fast spin echo (FSE) acquisition (3500/120 TR/TE) for anatomic and tumor delineation, and a proton density 3D True Fast Imaging with Steady State Precession imaging (CE-TrueFISP – 4.7/2.4 TR/TE) for optimal marker visualization. Both image sets are acquired in the same axial orientation with 26 slices (3 mm thickness) and a field of view of 20 × 20 cm. The images are then superimposed and a reference MR image is created by identifying the marker locations on the anatomic T2-weighted FSE images.

Non-contrast treatment planning CT images are acquired with 3 mm slice thickness and a field of view of 48 cm to encompass the skin surface. For both MRI and CT treatment planning image acquisitions, patients empty their bladders and are positioned supine with no knee support and their feet bound together. The reference MR images are then rigidly co-registered to the CT images (AcQSim, Philips Medical Systems, Netherlands) by identifying the common fiducial marker locations. The clinical target volume (CTV), which most commonly comprises the prostate gland, is defined on the co-registered reference MR image. The seminal vesicles, the rectum, and the streaking artifact from the fiducial markers are segmented on the CT images. Note that bloom artifact from the fiducial markers was similarly present on MR images.

A margin of 1.5 cm radial and 1 cm posterior is added to the CTV to generate the PTV for the first phase of the treatment. After 46Gy, the margin is reduced to 1 cm radial and 0.7 cm posterior, consistent with standard of care without daily image verification [8]. The total dose delivered ranges from 70–74Gy. A treatment plan is generated with a four-field technique to encompass the PTV with the 98–100% isodose. The radiation dose is prescribed to the 100% isodose. Digitally reconstructed radiographs (DRRs) with the overlying MLC profile and fiducial marker outlines are generated, electronically saved, and printed for reference to the portal films acquired on the first day of treatment.

#### Daily verification and correction of isocenter position

Isocenter placement relative to fiducial markers is verified on a daily basis prior to radiation delivery using an EPID on the linear accelerator (Clinac ^® ^21EX-Varian). With the patient in the treatment position, anterior-posterior and left lateral electronic images are acquired with an amorphous silicon (a-Si) flat panel EPID. A single portal image exposure is acquired using the treatment field's MLC profile and energy. The fiducial markers are clearly visible using 5 and 7 monitor units (MU) for the AP and lateral portal images respectively. This portal imaging dose is included in the daily treatment dose delivery.

**Figure 1:**
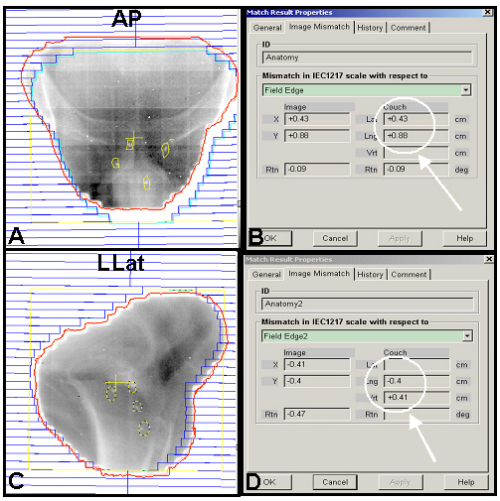
Illustration of software interface for manual matching of fiducial markers. Panels A and C show portal images (anterior-posterior (AP) and left lateral (LLat) respectively, red MLC profile) with a superimposed diagram representing the treatment planning MLC (blue profile) relative to fiducial markers locations (yellow outline). The therapist has manually aligned the yellow marker outlines in the treatment planning diagram to the radiopaque markers in the portal image. Panels B and D (corresponding to panels A and C, respectively), represent the magnitude of couch movement required for a match (arrow). Using a threshold of 5 mm, a longitudinal shift (inferiorly) of 9 mm was required.

Treatment planning MLC profiles with the relative fiducial marker outlines are sent to the Vision™ software (Varian) for comparison with the daily portal images. We assign the property of the marker outlines to "matching anatomy" and a field aperture is created. After each portal image is acquired, the radiation therapist uses the *Anatomy Match *function on the *Review *workspace in Vision™ to manually translate and align the yellow reference fiducial marker outlines to the radiopaque markers on the portal image. (Figure 1) Note that the yellow outlines are larger than the radiopaque markers due to streaking artifact on CT images. For simplicity no rotation is permitted in this alignment. The magnitude of the orthogonal vectors which comprise the 2D sum vector distance between the planned and actual marker location is then automatically calculated and displayed for the x and y dimensions. In our standard supine, head-first patient set-up, the x dimension represents left-right in anterior-posterior (AP) images and anterior-posterior in left lateral images. The y dimension is the superior-inferior direction for all images. If the distance is less than 5 mm, the treatment is delivered. Otherwise, the patient is repositioned and re-imaged for verification until the distance is less than 5 mm. Radiation therapists are responsible for documenting the shifts on a standardized form. After gaining experience with the first 83 consecutive treatments, the threshold for repositioning was reduced to 3 mm.

#### Study design- determination of intra and inter-therapist reproducibility

Datasets for 10 treatments (10 AP and 10 Left Lateral portal images) in each of two patients were archived for this study, for a total of 40 images. Radiation therapists familiar with the daily verification technique were instructed to manually align the fiducial markers and document the two absolute orthogonal shift distances for all 40 images, for a total of 80 measurements. Therapists were instructed to perform this task independently. This exercise was repeated, within one to two days, by each of the four therapists. Data were tabulated, and the intra and inter-radiation therapist variability was calculated with simple descriptive statistics, including the mean, median, and standard deviation of the data. The data were analyzed in terms of absolute difference between any pair of alignments. Inter-radiation therapist variability analysis was therefore based on 1920 data points, derived from 24 comparison datasets of 80 points each (6 comparisons for each of 4 radiation therapists).

## Results

**Figure 2:**
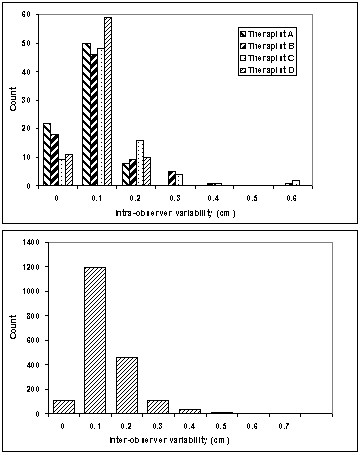
Intra-observer (A) and inter-observer (B) variability. Histograms depict the distribution of magnitude differences withinand between each therapist's measurements in the manual match technique.

The mean and median intra-observer error of the measured distance for the manual match were 0.4 and 0.3 mm (SD 0.5 mm) for observer A, 0.7 and 0.4 mm (SD 0.9 mm) for observer B, 0.5 and 0.5 mm (SD 0.4 mm) for observer C, and 0.9 and 0.6 mm (SD 1 mm) for observer D. (Figure 2A) Inter-observer results were similar with a mean error of 0.9 mm, a median of 0.6 mm, and a standard deviation of 0.7 mm. (Figure 2B) When using a 5 mm threshold, only 0.5% of treatments would undergo a table shift due solely to intra or inter-observer error in this study. If this threshold were reduced to 3 mm, 2.4% of table shifts would be due to observer error.

A very small but statistically significant difference was found in observer variability between lateral and AP portal image manual matches (AP mean 0.8 mm [CI 0.75–0.84], LLAT mean 1 mm [CI 0.94–1.1], P < 0.01).

This technique has now been clinically applied in 166 consecutive treatments in 6 patients. For the first 83 treatments, with a repositioning threshold of 5 mm, 30 treatments required table shifts prior to radiation delivery (36%). For the latter 83 treatments, with a threshold of 3 mm, 25 fractions required table shifts (30%). Approximately 5–10 minutes were dedicated to this verification depending on the need to reposition the patient.

## Discussion

With the advent of IMRT and highly conformal radiotherapy, there is mounting incentive to improve daily set-up and targeting accuracy of the prostate gland. Strategies to date have focused on reducing inter-fractional set-up error, and include alternative immobilization techniques[9], daily portal verification of isocenter position relative to bony landmarks [10], trans-abdominal ultrasound-based verification of prostate position relative to CT treatment planning contours (B-mode Acquisition and Targeting System -BAT^®^) [11], daily CT scans on the treatment couch [12], cone-beam CT mounted on the treatment gantry [13], and daily portal verification of fiducial marker locations relative to isocenter position [6,14,15]

In this study, we investigated the inter and intra-radiation therapist reproducibility in fiducial marker alignment using the "manual match" technique herein described. To our knowledge, there are no prior studies addressing this question. A review of the literature found two papers addressing intra and/or inter-user variability with trans-abdominal ultrasound for daily prostate positioning (BAT^®^). In Langen et al. [16], inter-user variability of the BAT^® ^system was investigated with eight users, including 4 radiation oncologists, 2 physicists, 1 urologist, and only 1 radiation therapist. A variability of greater than 2 mm was found in 50%, and greater than 4 mm in 25% of cases. Using the same system, Serago et al. [11] found inter-user variability to be greater than 3 mm in approximately 10% of measurements, and intra-user variability was greater then 3 mm in approximately 5% of cases depending on the orientation of shift. Limitations of the BAT^® ^system which may account for it's poor inter and intra-user reproducibility include error in the initial CT and isocenter definition of the BAT^® ^test phantom, and uncertainties in the CT definition of the prostate which translate directly into a systematic uncertainty in the BAT^® ^alignment [16].

Using the fiducial marker technique, we achieved superior results with an observer variability of greater than 3 mm observed in only 2.4% of cases. Furthermore, this was found with four radiation therapists involved in the routine treatment of our patients. Another advantage of the fiducial marker approach is that it is not dependent on the location of the prostate gland relative to the pubic symphysis [16] and is less dependent on patient size and weight [17]. The variability we observed may in part be due to uncertainty in the manual alignment as the marker outline is larger than the radiopaque marker visualized on the portal image. Prior studies have shown that the markers do not migrate significantly during a course of therapy, and as such, are reliable surrogates to the position of the prostate gland [18,19]. The technique does not require specialized localization software or hardware modifications beyond standard portal image software. It permits portal imaging to be limited to the treatment field for daily localization, sparing surrounding normal tissues from cumulative dose which would be delivered in alternative open field localization systems [19].

We have also found a very small (0.2 mm) but statistically significant increase in variability with the lateral alignment compared to the AP alignment. Although this difference is not clinically significant and is smaller than the pixel size of the EPID (0.8 mm), it may point to poorer visualization of the fiducial markers on the lateral image, or to greater difficulty in alignment due to rotation of the prostate gland along this axis.

There are limitations of our study design. We did not address the radiation therapist's accuracy in the actual table shift at the second verification. There was also no systematic assessment of time cost to this procedure on a daily basis. Finally, the four radiation therapists had a relatively short interval of one to two days between the two measurements. A larger user error might have been found by increasing this interval.

Despite our reported level of accuracy, we acknowledge that intra- and inter-radiation therapist variability is not the sole source of set-up error in this technique. For simplicity, we have opted to ignore rotational errors in alignment at the inception of this trial. Others have introduced a collimator rotation in the lateral treatment fields if the required rotation angle exceeds 3 degrees [6]. Future work will determine the need, feasibility and reproducibility of such a correction, as well as an assessment of the important impact of intra-fractional organ and patient motion. This work will be necessary in order to determine whether PTV margins can be safely reduced with this technique.

In conclusion, we have found high intra and inter-radiation therapist reproducibility with a simple method for daily verification and correction of isocenter position relative to fiducial markers using electronic portal imaging. We believe this is an important first step toward an eventual goal of PTV reduction and safe dose escalation.
